# Care-seeking and health insurance among pregnancy-related deaths: A population-based study in Jember District, East Java Province, Indonesia

**DOI:** 10.1371/journal.pone.0257278

**Published:** 2022-03-23

**Authors:** Trisari Anggondowati, Poppy E. Deviany, Kamaluddin Latief, Annis C. Adi, Fitri Nandiaty, Anhari Achadi, Henry D. Kalter, Emily H. Weaver, Tika Rianty, Mahlil Ruby, Sri Wahyuni, Akhir Riyanti, Naintina Lisnawati, Nissa Kusariana, Endang L. Achadi, Philip W. Setel

**Affiliations:** 1 Center for Family Welfare, Faculty of Public Health, Universitas Indonesia, Depok, Indonesia; 2 Faculty of Public Health, Universitas Airlangga, Surabaya, Indonesia; 3 Institute for International Programs, Department of International Health, Johns Hopkins Bloomberg School of Public Health, Baltimore, MD, United States of America; 4 Carolina Population Center, The University of North Carolina at Chapel Hill, Chapel Hill, North Carolina, United States of America; 5 USAID Jalin Project, Indonesia implemented by DAI Global LLC, Jakarta, Indonesia; 6 Faculty of Public Health, Universitas Diponegoro, Semarang, Indonesia; 7 Vital Strategies, New York, NY, United States of America; Flinders University, AUSTRALIA

## Abstract

**Background:**

Despite the increased access to facility-based delivery in Indonesia, the country’s maternal mortality remains unacceptably high. Reducing maternal mortality requires a good understanding of the care-seeking pathways for maternal complications, especially with the government moving toward universal health coverage. This study examined care-seeking practices and health insurance in instances of pregnancy-related deaths in Jember District, East Java, Indonesia.

**Methods:**

This was a community-based cross-sectional study to identify all pregnancy-related deaths in the district from January 2017 to December 2018. Follow-up verbal and social autopsy interviews were conducted to collect information on care-seeking behavior, health insurance, causes of death, and other factors.

**Findings:**

Among 103 pregnancy-related deaths, 40% occurred after 24 hours postpartum, 36% during delivery or within the first 24 hours postpartum, and 24% occurred while pregnant. The leading causes of deaths were hemorrhage (38.8%), pregnancy-induced hypertension (20.4%), and sepsis (16.5%). Most deaths occurred in health facilities (81.6%), primarily hospitals (74.8%). Nearly all the deceased sought care from a formal health provider during their fatal illness (93.2%). Seeking any care from an informal provider during the fatal illness was more likely among women who died after 24 hours postpartum (41.0%, OR 7.4, 95% CI 1.9, 28.5, p = 0.049) or during pregnancy (29.2%, OR 4.4, 95% CI 1.0, 19.2, p = 0.003) than among those who died during delivery or within 24 hours postpartum (8.6%). There was no difference in care-seeking patterns between insured and uninsured groups.

**Conclusions:**

The fact that women sought care and reached health facilities regardless of their insurance status provides opportunities to prevent deaths by ensuring that every woman receives timely and quality care. Accordingly, the increasing demand should be met with balanced readiness of both primary care and hospitals to provide quality care, supported by an effective referral system.

## Introduction

Indonesia, the world’s fourth most populous country, faces significant challenges in reducing maternal mortality [1]. The country’s maternal mortality ratio (MMR) of 305 per 100,000 live births in 2015 remains among the highest in the region [2, 3]. The high MMR poses a big challenge in achieving Sustainable Development Goal 3 (SDG-3) which aims to reduce the global MMR to less than 70 per 100,000 live births by 2030, with no greater than 140 per 100,000 live births in any country [4]. This situation calls for concerted action to catalyze progress toward achieving the SDG-3 targets.

Reducing maternal mortality requires a good understanding of the magnitude of the problem, causes, and underlying factors. Notably, given the increase in facility-based deliveries in Indonesia from 46% to 74% between 2007 and 2017 [5, 6], it is important to understand the care-seeking pathways that women take to shed light on why maternal mortality remains high. Social factors related to maternal health care utilization and outcomes include sociocultural beliefs and practices, lack of awareness about maternity care and danger signs in pregnancy, and household decision-making dominated by husbands and other family members, as shown by qualitative studies in Indonesia [7, 8]. Two large-scale cohort studies in Bangladesh show that only between 29% and 47% of women who experienced obstetric complications received care from trained providers, with household wealth, women’s and husbands’ education, and distance to health facility among the predictors for care-seeking [9, 10]. Studies from low- and middle-income countries, including Indonesia, indicate that cost of services has also been a barrier in access to health care, especially for the poor [11, 12].

To ensure access to safe, effective, quality, and affordable healthcare for all, as mandated by SDG-3, the Government of Indonesia has implemented the National Health Insurance (NHI) policy (*Jaminan Kesehatan Nasional/*JKN) since 2014, to move toward universal health coverage (UHC) [13]. By 2018, when this study was conducted, nearly 80% of the population were covered by the NHI [14]. Those who do not have any insurance and are not covered by the NHI, but are classified as poor or near-poor, may receive central and/or local government aid [15]. The rapidly growing NHI coverage, combined with the other sources of government assistance, may have successfully alleviated financial barriers for maternal and neonatal health services, although inequity in access persists particularly by different economic levels [16]. Furthermore, the influence of improved health insurance coverage on care-seeking for complications that led to pregnancy-related deaths is not well-known. Previous studies on maternal health care-seeking in the Indonesian setting were mostly done prior to, or at the early stage of, the NHI roll-out [16, 17].

A good understanding of care-seeking patterns related to complications is important to inform effective interventions to help prevent avoidable deaths in the future, yet the necessary information remains lacking. This study aimed to fill the gap by providing information about the causes of death and care-seeking pathways leading to maternal mortality, and to examine whether patterns of care-seeking vary by health insurance status.

## Materials and methods

### Study design

This paper used a subset of data from a large cross-sectional study on maternal and neonatal mortality, namely the ‘Every Mother and Newborn Counts’ (EMNC) study, with district-wide coverage, conducted from January to April 2019. The EMNC study was undertaken as part of the five-year USAID Jalin project [18], which aims to reduce maternal and neonatal mortality in Indonesia. Data on maternal mortality were obtained from two methods of data collection: i) the ‘Maternal deaths from Informants and Maternal deaths Follow on Review’ (MADE-IN/MADE-FOR) to identify pregnancy-related deaths [19], and ii) a Verbal and Social Autopsy (VASA) interview to ascertain the circumstances and causes of death [20]. The VASA tool integrates the 2016 World Health Organization Verbal Autopsy instrument with the Johns Hopkins University/Institute for International Programs Social Autopsy questionnaire to collect information on the medical and injury history associated with final illness, general signs and symptoms associated with the final illness, pregnancy and delivery complications, care-seeking prior to death, and household characteristics [20].

### Study setting

The EMNC study was conducted in Jember District on the island of Java, home to about 2.4 million people. The study covered all 248 villages in the district [21]. The study district was selected due to the high number of maternal deaths, the large population, and the geographical and socio-cultural challenges in reducing maternal mortality. The district has reported relatively good maternal health care status as exhibited by the proportion of government-recommended antenatal care visits attended (88.5%) and deliveries in facilities (91.2%). There are 11 general hospitals in the district, of which four are Comprehensive Emergency Obstetric and Neonatal Care (CEmONC) facilities [22]. At the time of the study, nearly 60% of the population in the study district were registered under the NHI, of whom 45.5% were financed by the local government [22].

### Study population

The study population was women of reproductive age (WRA), aged 13–49 years old, who died while pregnant, during labor/delivery, or within 42 days after delivery or abortion, irrespective of the cause of death. The eligibility criteria were women who were residents (proven with identity card) or who have stayed for at least six months in the study district who died between January 2017 and December 2018.

### Data collection

The MADE-IN/MADE-FOR method sought to identify all pregnancy-related deaths through a listing of WRA deaths in each village. The list, with information on whether the death occurred during pregnancy, labor/delivery, or within 42 days after delivery or abortion, was used to screen for possible pregnancy-related deaths. The listing was produced by obtaining information from two networks of village informants who are considered most knowledgeable of the vital events in the community. Details of the method are described elsewhere [19, 23]. All households identified as having experienced a pregnancy-related death during the recall period were visited to verify eligibility. Once deaths were confirmed as eligible, a VASA interview was conducted [20]. The VASA was held by interviewing household respondents, primarily family member(s) who were considered as the most knowledgeable about circumstances and care-seeking actions taken during the fatal illness. A two-year recall period (2017–2018) was used to increase the likelihood of obtaining sufficient events for robust analyses while minimizing recall bias.

The term formal care provider refers to a trained or certified health care provider (i.e. doctor/Obstetrician/Gynecologist, midwife, and nurse) regardless of the place where care was provided (inside or outside health facilities). Informal care provider refers to the individual providing traditional medicines, home remedies, or non-professional treatment. We differentiated between the first and last care provider seen/visited/sought (even if the deceased did not reach the provider) during the fatal illness before death. We categorized health insurance status as (1) insured (NHI or other health insurance type), (2) government aid beneficiaries, and (3) uninsured. Government aid refers to either *Jaminan Kesehatan Daerah/Jamkesda* (Regional Health Insurance), which is provided through the local government financing and can be used for any type of care, or *Jaminan Persalinan/Jampersal* (Social Insurance for Free Delivery), which is financed by the central government and designated only for maternal and neonatal care. A proxy of the socio-economic level was measured through wealth quartiles, with Quartile 1 being the poorest and Quartile 4 the least poor. The wealth quartiles are a composite of household assets and the index was derived using principal components analysis.

### Data management and quality assurance

Data were collected and directly entered on a tablet with the Open Data Kit (ODK) platform, and then transmitted electronically to the central research office for storage, management, cleaning, and analysis. Before being transmitted to the central office, data were cross-checked by a different data collector and then double-checked by the field supervisor. Transmitted data were also checked by the data management team as part of the data cleaning.

### Data analysis

Data from the ODK were transferred into STATA for analysis. We analyzed the number of pregnancy-related deaths and their characteristics using descriptive statistics. Bivariate analysis was conducted to identify any distinct patterns of care-seeking that existed by health insurance status, cause of death, and other variables. Information from the VASA was analyzed to determine the causes of death, identify social factors contributing to deaths, and examine care-seeking pathways. The causes of death were assigned by the InSilicoVA computer algorithm [24]. For each death, InSilicoVA assigns the most likely underlying cause.

### Ethical approval

The study was granted ethical approval from the Institutional Review Board at the Biomedical Research Alliance of New York (BRANY) and at the Faculty of Public Health, Universitas Indonesia. Written informed consent was obtained from each respondent prior to participating, and each respondent was also provided a hard-copy description of the study and a consent statement with the local Principal Investigator’s contact information.

## Results

**Fig 1:**
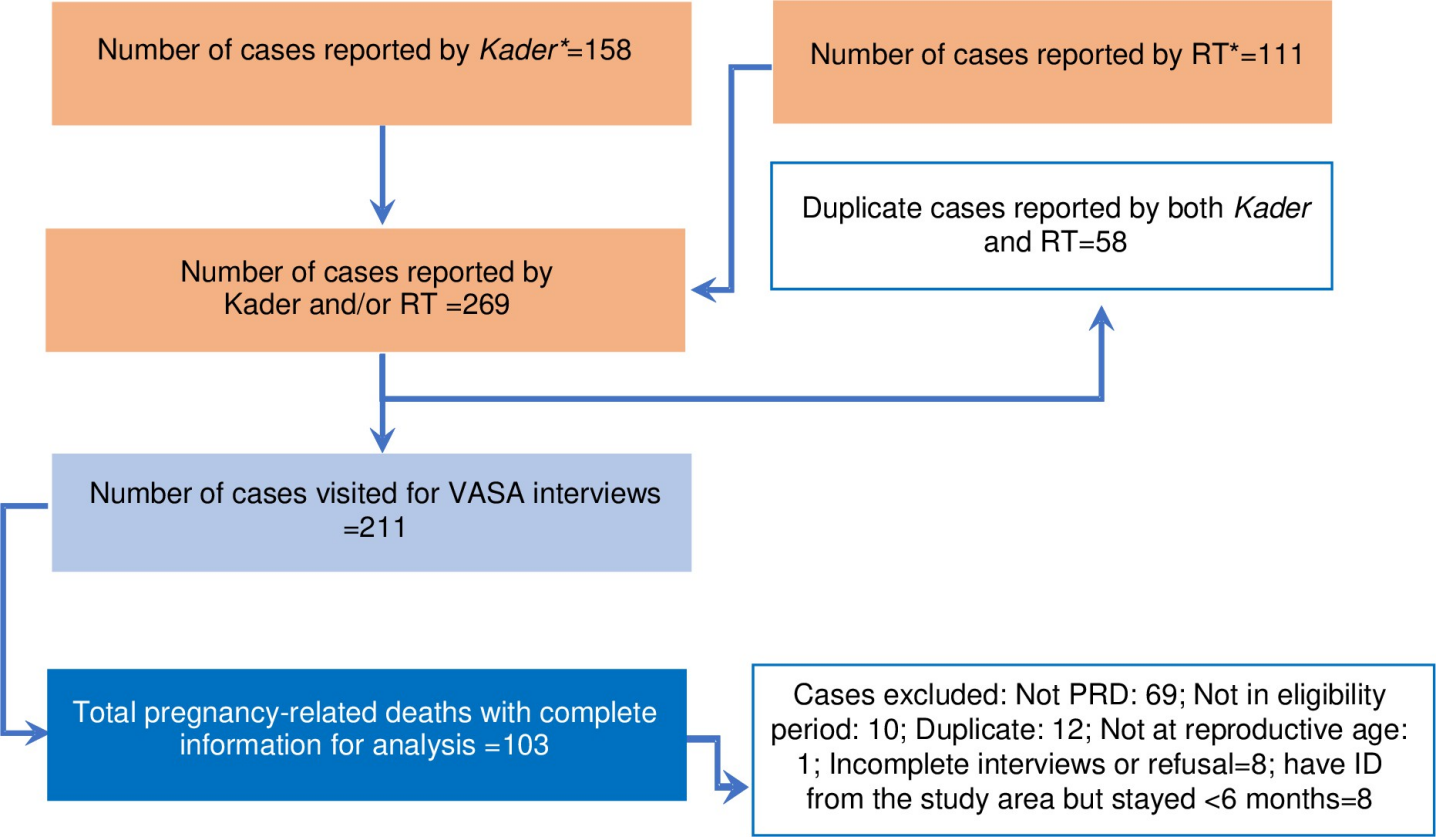
Flowchart of pregnancy-related death (PRD) cases identified in Jember District, 2017–2018. *Kader: community health volunteer; *RT*: head of a neighbourhood unit. These two groups are generally the most knowledgeable about vital events in the community, as shown from the previous implementation of the MADE-IN/MADE-FOR methods.

Among 269 WRA deaths reported, 211 deaths were listed as pregnancy-related deaths by the village informants. After confirmation with families of the deceased, 48.8% of the cases met our inclusion criteria and the VASA interviews were completed. The remaining (n = 108) were deemed ineligible due to i) not being pregnancy-related deaths (63.9%); ii) died outside of the 2017–18 recall period (9.3%); iii) duplicate cases (11.1%), and other reasons (Fig 1). We also matched the cases found in our study against the district’s health information system, which collects information on maternal deaths (excluding deaths due to accidental or incidental causes), and found that our study identified 26% more maternal deaths. The final analysis for this study included 103 eligible pregnancy-related deaths. The researchers decided to focus on pregnancy-related deaths in this study and included the three deaths due to accidental causes. All data presented in the tables and the figure in this section were generated from the VASA interviews.

### Descriptive characteristics of pregnancy-related deaths

Table 1 describes the characteristics of the pregnancy-related death cases. The mean age at death was 31 years (±7), with about one-third occurring among women aged 35 years or older. About 47% of the women were first married when less than 20 years old. The greatest number of pregnancy-related deaths occurred among women with no education or primary education only (41.8%), living in rural areas (55.3%), and of Madura ethnicity (61.2%). About one-third of the women were primiparous (28.2%) and 9.7% had four or more previous births.

**Table 1:** Characteristics of pregnancy-related death cases (n = 103) in Jember District, 2017–2018.

**Characteristics**	**n (%)**
**Age at death (years)**	
15–19	6 (5.8)
20–24	18 (17.5)
25–29	19 (18.4)
30–34	25 (24.3)
35–39	22 (21.4)
40+	13 (12.6)
**Education**	
None or Primary	43 (41.8)
Junior High	27 (26.2)
Senior High	20 (19.4)
Academy/University	9 (8.7)
Don’t know	4 (3.9)
**Age when first married (years)**	
< 16	9 (8.7)
16–19	39 (37.9)
≥ 20	43 (41.7)
Don’t know	12 (11.7)
**Prior births**	
0	29 (28.2)
1	29 (28.2)
2	28 (27.1)
3	7 (6.8)
4 and more	10 (9.7)
**Residence**	
Urban	46 (44.7)
Rural	57 (55.3)
**Ethnicity**	
Javanese	40 (38.8)
Madura	63 (61.2)
**Place of delivery (among women who died during or after delivery) (n = 75)**	
Hospital	51 (68.0)
Other health facility	10 (13.3)
Enroute to health facility	8 (10.7)
Home	1 (1.3)
Other	5 (6.7)
**Place of death**	
Hospital	77 (74.8)
Primary health facility	7 (6.8)
Enroute to health facility	10 (9.7)
Home	9 (8.7)
**Time of death**	
Pregnancy	25 (24.3)
Delivery or within the first 24 hours after delivery	37 (35.9)
2–7 days postpartum	15 (14.6)
8–15 days postpartum	10 (9.7)
16–42 days postpartum	16 (15.5)
**Insurance**	
Insured (NHI or other insurance scheme)	55 (53.4)
Government aid	24 (23.3)
Uninsured	24 (23.3)
**Causes of deaths**	
Obstetric haemorrhage	40 (38.8)
Pregnancy-induced hypertension	21 (20.4)
Pregnancy-related sepsis	17 (16.5)
Other and unspecified cardiac disease	12 (11.7)
Other causes*	13 (12.6)

*Others include abortion-related death (n = 1), anemia of pregnancy (n = 1), and digestive neoplasms (n = 1), diarrheal diseases (n = 1), liver cirrhosis (n = 1), accidental exposure to fire (n = 1), road traffic accident (n = 2), other and unspecified maternal causes (n = 4), and other and unspecified cause of death (n = 1).

The highest percentage of pregnancy-related deaths occurred more than 24 hours postpartum (40%), followed by deaths at delivery or within 24 hours postpartum (36%) and during pregnancy (24%). Among women who died during or after delivery, 81.3% delivered in health facilities, including 68% in hospitals and 13.3% in lower-level facilities. About 82% of all pregnancy-related deaths occurred in health facilities, with the majority in hospitals (74.8%) and the remainder in primary health facilities (6.8%).

### Causes of death

The three leading causes of pregnancy-related death were obstetric hemorrhage (38.8%), pregnancy-induced hypertension (PIH) (20.4%), and pregnancy-related sepsis (16.5%) (Table 1). We examined cause of death in relation to pregnancy, labour and delivery. Among women who died during pregnancy, about one-third were due to PIH (32%), followed by pregnancy-related sepsis (28%). Most deaths of women who died during labour/delivery or within the first 24 hours postpartum were due to obstetric hemorrhage (78.4%), followed by PIH (10.8%). Hemorrhage, PIH, and pregnancy-related sepsis were equally represented as causes of death among women who died more than 24 hours postpartum; each contributed 22% of the deaths (S1 Table).

### Care-seeking pathways

**Fig 2:**
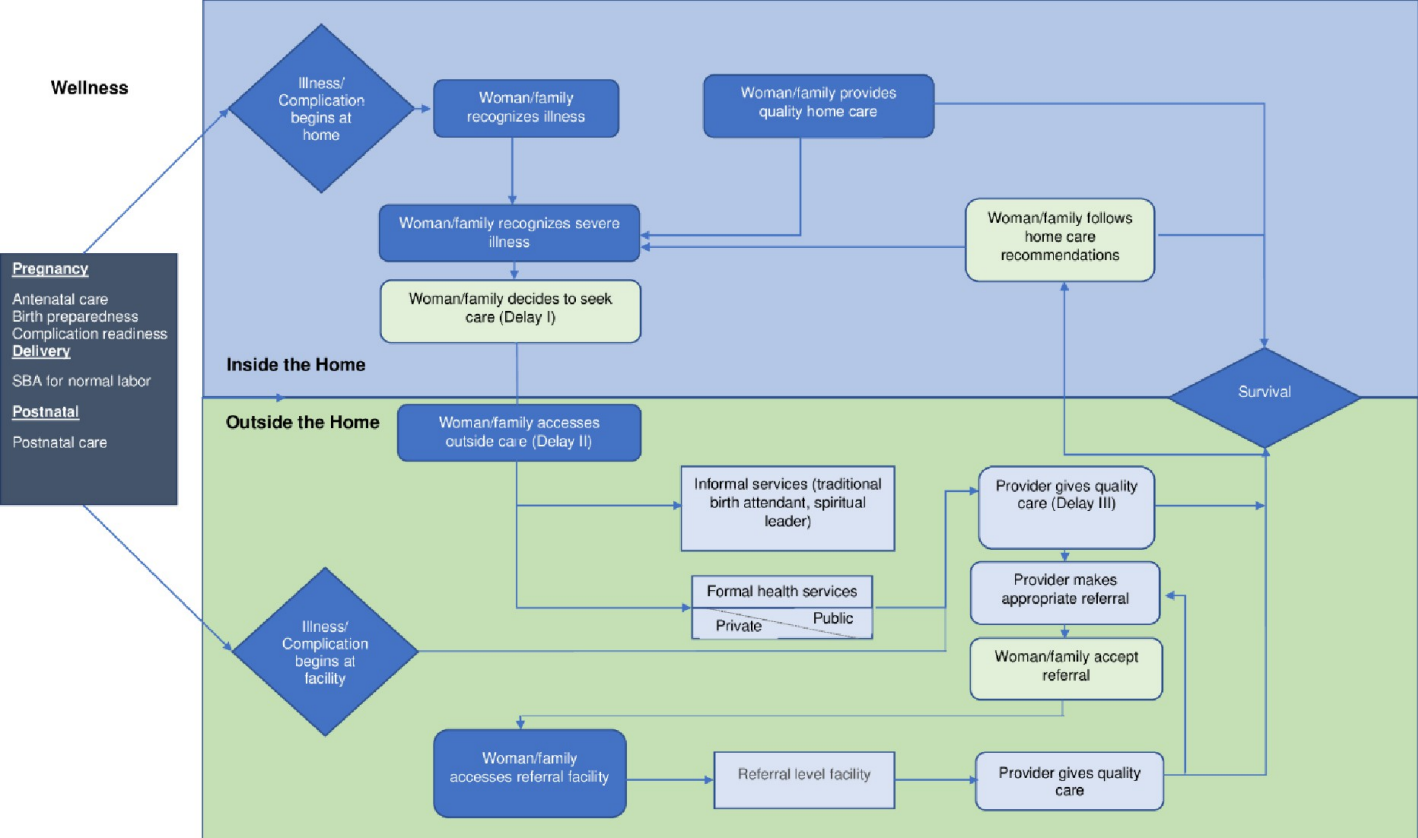
Care-seeking pathways among pregnancy-related deaths. This pathway was adapted from the Pathway to Survival for children developed to support the WHO/UNICEF Integrated Management of Childhood Illness approach [25].

Fig 2 shows the care-seeking pathway that women may traverse during their illness prior to death. The figure was adapted from the Pathway to Survival for children developed to support the WHO/UNICEF Integrated Management of Childhood Illness approach [25]. The adapted pathway includes different points of entry for women based on where their illness began. Table 2 summarizes steps in the care-seeking pathway among all pregnancy-related deaths, stratified by time of death and insurance status. Nearly all pregnancy-related deaths in our study (95.1%) sought care at some time during the fatal illness, including 93.2% that sought formal care (either alone or a combination with informal care). Among those who reached the first formal care provider, 65.9% were referred by the first health provider. Almost all of the deceased (96.4%) complied with all the referrals made by all formal providers seen during the illness.

**Table 2:** Summary of care-seeking pathways by time of death and insurance in Jember District, 2017–2018.

Factor	Care-seeking pathway								
	Sought any care	Sought formal care only*	Formal and informal care*	Informal care only*	OR (95% CI) p-value (any informal care vs. formal care only)^†^	Reached 1st formal provider^‡^	Referred, among those who reached 1^st^ formal provider	Complied with all referrals^‡^	Reached last formal provider, among those who were referred^‡^
	n (%)	n (%)	n (%)	n (%)	n (%)	n (%)	n (%)	n (%)	n (%)
All deaths (n = 103)	98/103 (95.1)	72/98 (73.5)	24/98 (24.5)	2/98 (2.0)		91/91 (100)	60/91 (65.9)	54/56 (96.4)	54/56 (96.4)
Time of death									
Pregnancy (n = 25)	24/25 (96.0)	17/24 (70.8)	7/24 (29.2)	0/24 (0)	4.4 (1.0, 19.2) 0.003	22/22 (100)	18/22 (81.8)	16/17 (94.1)	16/17 (94.1)
At delivery or within 24 hr postpartum (n = 37)	35/37 (94.6)	32/35 (91.4)	3/35 (8.6)	0/35 (0)	*Reference*	34/34 (100)	21/34 (61.8)	18/19 (94.7)	18/19 (94.7)
More than 24 hr postpartum (n = 41)	39/41 (95.1)	23/39 (59.0)	14/39 (35.9)	2/39 (5.1)	7.4 (1.9, 28.5) 0.049	35/35 (100)	21/35 (60.0)	20/20 (100)	20/20 (100)
Insurance ownership									
Insured (n = 55)	52/55 (94.5)	37/52 (71.2)	13/52 (25.0)	2/52 (3.8)	1.4 (0.4, 4.4) 0.589	46/46 (100)	29/46 (63.0)	28/29 (96.6)	28/29 (96.6)
Government aid (n = 24)	24/24 (100)	18/24 (75.0)	6/24 (25.0)	0/24 (0)	1.1 (0.3, 4.4) 0.857	24/24 (100)	17/24 (70.8)	14/15 (93.3)	14/15 (93.3)
Uninsured (n = 24)	22/24 (91.7)	17/22 (77.3)	5/22 (22.7)	0/22 (0)	*Reference*	21/21 (100)	14/21 (66.7)	12/12 (100)	12/12 (100)

*among those who sought care

^**†**^any informal care is a combination of seeking informal care only and both formal and informal care

^**‡**^excluded those who died prior to reaching 1^st^ provider, died before being referred, or died before reaching the last provider.

Of all deceased women who sought care from a formal provider, the decision to seek formal care was made by the woman’s spouse in nearly half of the cases (47.9%), followed by other family members or relatives (18.8%). Decision-making by the deceased herself or jointly with the husband occurred in about 18% of the cases. Only 13.5% of respondents (13 out of 96 cases) reported facing constraints when seeking care for the deceased. The most frequently mentioned constraints were the thought that the woman was too sick to travel (35.7%; n = 5) and dissatisfaction with the available health care (21.4%; n = 3). Other constraints mentioned by a smaller number of respondents include the perception that the woman was not sick enough to need care, that it was too far to travel, and others. Distance appears to have been the major factor influencing the choice of the first provider, as 56.3% of respondents mentioned that they chose providers who are nearby. Many other respondents chose providers who they were familiar with (21.9%), or felt safe with (15.6%) (Table 3).

**Table 3:** Care-seeking among pregnancy-related death cases who sought formal care (n = 96) in Jember District, 2017–2018.

Care-seeking	n (%)
**Decision maker for care-seeking (n = 96)**	
Adult deceased’s partner/spouse	46 (47.9)
Relatives	18 (18.8)
Adult deceased herself	9 (9.4)
Both deceased and her spouse	8 (8.3)
Health provider	8 (8.3)
Someone else	5 (5.2)
Don’t know	2 (2.1)
**Stated that there is concern in care-seeking (n = 96)**	
Yes	13 (13.5)
No	79 (82.3)
Don’t know	4 (4.2)
**Constraints in care-seeking (n = 13)**	
Too sick to travel	5 (35.7)
Not satisfied with available healthcare	3 (21.4)
Did not think she was sick enough	1 (7.1)
Too much time from her/caregiver’s duties	1 (7.1)
Too far to travel	1 (7.1)
Cost (transport, healthcare, other)	1 (7.1)
Other issue*	3 (21.4)
**Factors influencing choice of first provider (n = 96)** ^#^	
Provider nearby	54 (56.3)
Familiarity	21 (21.9)
Feel safe	15 (15.6)
Recommended by doctor/midwife	12 (12.5)
Comfortable	11 (11.5)
Medical reason (abnormality)	10 (10.4)
Modern services	7 (7.3)
Few choices	5 (5.2)
Cheap provider	4 (4.2)
Family reason	3 (3.1)
Other**	8 (8.3)

^#^multiple answers allowed

*e.g. afraid of caesarean section procedure

**includes perceived more timely or better care, better land road to reach the provider, and provider already designated by the referral system.

#### Care-seeking pathway by health insurance status and time of death

Table 2 does not show a distinct care-seeking pattern across insurance groups. Although the proportion of those who sought care was lowest among the uninsured group (91.7%), the difference with the insured group (94.5%) was negligible. Also, among the uninsured group who were referred, all complied with the referral. However, the data show differences in the care-seeking pattern by pregnancy status at time of death. Women who died more than 24 hours postpartum were most likely to seek any care from an informal provider (41.0%, OR 7.4, 95% CI 1.9, 28.5, p = 0.049), followed by women who died during pregnancy (29.2%, OR 4.4, 95% CI 1.0, 19.2, p = 0.003), compared to those who died during delivery or within 24 hours postpartum (8.6%) (ORs and 95% CIs are not presented in the table). The proportion of seeking any care from an informal provider is the sum of those who sought both formal and informal care, and those who sought informal care only.

### Health insurance

While 76.7% of the deceased had insurance coverage, only 44.8% used insurance at the first point of contact with a formal care provider. Table 4 shows that among deceased who were insured or received government aid and went to more than one provider (n = 55), 54.5% and 92.7% used insurance at the first and last provider, respectively. Women who had insurance were mixed by their wealth quartile, without a distinct pattern toward either the wealthy or the poor group. The proportion of women who died in health facilities was consistently high across all insurance groups. While a higher proportion of women with insurance or government aid died in a hospital than did uninsured women, the difference was not statistically significant (p = 0.110) (S2 Table).

**Table 4:** Health insurance use among insured or government aid beneficiaries who went to multiple providers (n = 55).

Use of health insurance		At the last care provider		Total
		Yes	No	n (%)
At the first care provider	Yes	29	1	30 (54.5%)
	No	22	3	25 (45.5%)
Total		51 (92.7%)	4 (7.3%)	55 (100%)

## Discussion

This study is among the few population-based studies of pregnancy-related mortality in Indonesia that sought to capture data representative at the district level. It is also the first to apply the VASA tool to determine the cause of death and to analyze relevant health system-related and social factors of pregnancy-related death in Indonesia. Using the MADE-IN/MADE-FOR method, this study identified 26% more cases of maternal deaths than the district’s routine health information system, which increases our confidence in the completeness of the data.

### Causes of death

The causes of death distribution in our study population is generally consistent with findings from other studies [26]. Our study, however, observed a higher percentage of deaths due to pregnancy-related sepsis (16.5%) compared to the global figure of 10.7% [26]. The literature suggests that the global figure may underestimate the contribution of infections to pregnancy-related deaths, as they do not include deaths due to abortion-related infections or non-obstetric infections that can be aggravated by pregnancy [27]. Furthermore, there is evidence that clinicians fail to recognize the signs and symptoms of possible infections [28, 29]. A cohort study conducted in 713 health facilities in 52 low-, middle- and high-income countries showed that at least one source of infection was identified for 79.7% of women hospitalized, with the most common sources of infections being the genital (endometritis and chorioamnionitis) or urinary tract, skin or soft tissues, respiratory tract, and abortion-related. In the study, infection was the underlying cause in more than half of the in-hospital deaths [27].

A more in-depth analysis of our data showed that a high percentage of the sepsis-related cases occurred among deaths during pregnancy and more than 24 hours postpartum. Our study, however, was not able to identify the common source of infection among the sepsis-related cases to inform recommendations for control measures. Nevertheless, the relatively high proportion of sepsis-related deaths in our study population, despite good access to care, raises concerns about quality of care, early identification, and management of sepsis, particularly during the antenatal and postpartum periods. This deserves further research.

### Care-seeking

Nearly all women in our study sought formal care at some time during the fatal illness, and almost all deceased complied with all the referrals made by the formal providers seen. A more in-depth analysis did not show a distinct pattern of the care-seeking pathway by insurance status. The uninsured group sought and received care from formal care providers and complied with referral recommendations to a similar degree as the insured group. Other studies, including those that include Indonesian data, found significant positive effects of health insurance coverage on maternal health care utilization [30, 31]. While several previous safety net programs designed to provide access to care for the poor were in place at the time of these studies, they predate the NHI era and efforts to expand universal access to care, which may explain the different findings.

Given that our study population of pregnancy-related deaths experienced life-threatening complications, the severity of these complications may have led the women/families to seek care regardless of their insurance status, as shown by the absence of differences in care-seeking across the insurance groups. The perceived severity of illness is one factor affecting health care utilization in some of the leading health behavior models [32, 33]. As a study from Bangladesh found, however, perceived severity can be mediated by concerns over medical costs [12]. Our study found that the proportion of deaths in the hospital was higher among the insured group and those who received government aid (78.2% and 79.2%, respectively) compared to the uninsured (62.5%), although not statistically significantly so. This finding may indicate that, despite the growing NHI, to some extent cost may still serve as a barrier especially in access to hospital care, consistent with the Bangladesh study [12].

Care-seeking from informal providers appeared to be influenced by pregnancy status. Women who died more than 24 hours postpartum or during pregnancy were more likely to seek care from an informal care provider at any time during the fatal illness, as opposed to those who died during delivery or within 24 hours postpartum. We hypothesize that the latter group may be perceived as at a greater threat than the other groups, and thus, the majority sought care directly from a formal care provider. Further examination of the causes of death appears to support this hypothesis, as about 78% of deaths during delivery or within the first 24 hours postpartum were attributable to obstetric hemorrhage. The bleeding and the fact that the women were undergoing or had just completed delivery may have triggered families to seek formal care. This premise is corroborated by a qualitative study in Jayawijaya District of Indonesia that found bleeding as a commonly recognized danger sign if present during pre-labor and that rapid decision-making usually occurred when visible symptoms of excessive bleeding began to appear [8]. PIH and pregnancy-related sepsis, which were associated with higher percentages of deaths during pregnancy and after 24 hours postpartum, may not be recognized as posing as big of a threat as bleeding. Also, once a woman has completed labour and delivery, attention may focus more on the newborn and she or her family may not consider that some complications can be life-threatening for the woman [34]. This lack of perceived urgency during many postpartum complications may have also contributed to the increased proportion of deaths during the postpartum period [34, 35]. If this is true, our findings have strong policy implications to increase the quality of postnatal care, one aspect of which would be to improve the identification and management of postnatal complications.

### Other factors related to care-seeking

Consistent with literature from developing countries, this study suggests the lack of autonomy among women in deciding to seek care, as less than 10% of the deceased participated in the decision-making during final illness. This finding highlights the need to empower women to enhance their decision-making authority, while at the same time educating women and their partners and families about birth preparedness and complication readiness. Our study found that the majority of the deaths occurred in one ethnic group, the Madura. Although certain cultural practices related to ethnicity are closely associated with maternal mortality [36], we cannot determine the effect of ethnicity in our study population. Given the fact that this ethnic group mostly resides in hard-to-reach areas, geographic challenges may complicate their care-seeking, and eventually have led to poorer outcomes.

Although our study was restricted to deaths, the fact that the majority of the women went to formal care and a relatively small proportion of respondents reported constraints when seeking care for the deceased should lead us to acknowledge at least the success of the health system in Indonesia in alleviating barriers to health care access.

### Health insurance

Improvement of the health insurance scheme in Indonesia has increased access to maternal health care, predominantly among the poor and those living in less-developed areas [37]. A shift of pregnancy-related deaths from home to health facilities was evident in our previous study comparing data over a 10-year period [35]. In the current study, most deliveries and deaths also occurred in health facilities. Cost was not mentioned as a significant constraint for care-seeking, contrary to prior VASA studies of pregnancy-related deaths [38]. However, our current findings concerning both the coverage and utilization of insurance by women dying from pregnancy-related causes in this district paint a complex picture. We found, for example, no clear pattern of insurance scheme participation associated with wealth quartiles; low usage of insurance at the first formal provider consulted; and that higher use of insurance when sought care at the last providers than at the first (when multiple providers were involved).

The low usage of insurance at the first formal provider may be explained by several factors including the possibility that the preferred provider may have been a non-NHI designated provider (common among private providers), or some participants may have found that the tiered referral system of the NHI was too complicated to navigate [39–41]. Under the NHI, referral in Indonesia applies a tiered system, in which the primary care provider is designated as the entry point for care and referral to specialized care if the primary care provider is unable to handle the case, except in an emergency [13], when patients can directly seek care from referral health facilities [15]. Another possible explanation is that when they reached the last care provider many women may have required more intensive care or longer hospitalization due to the illness progression. This may lead to catastrophic expenditure, and thus, to higher use of insurance or government aid.

### Study limitations

The major limitation of this study is that it only explored the experiences of women who died; there was no control group. Interpretation and generalization of results need to be made with caution. Determinants of care-seeking and maternal deaths cannot be established from the data and the findings cannot be generalized to the general population of women with obstetric complications. The retrospective nature of the data being gathered in this study may introduce recall bias. To minimize the bias, we limited our recall period to two years. However, since pregnancy-related death is a tragic event, it is more likely that family members would remember most of the details surrounding the deaths. Thus, we believe that recall bias does not affect the results and conclusions of this study. This study used data reported from interviews with family members of the deceased, and thus, information on the signs and symptoms were gathered according to the family’s perception, which may not be as accurate as the health providers’ evaluation. However, in general the causes of death distribution in this study is consistent with findings from others, and the relationship of the causes to the time of death is plausible. Also, this study was not designed to assess the quality of care at health facilities, and thus, we cannot draw conclusions about delays in receiving quality care.

This study provides a more comprehensive description of pregnancy-related deaths at sub-national level and adds to the current knowledge, particularly in the context of a high rate of facility-based deliveries and UHC. However, there are clearly several important areas that require better understanding to inform effective maternal mortality reduction strategies, which this study was unable to address. Quality of care and how health financing is functioning to improve health are clearly among the areas that call out for additional research. Our study indicates that access to care may no longer pose a major barrier, largely due to the increasing coverage of NHI. However, concerns over the quality of care remain, which NHI could not directly overcome. Implementing strategic health purchasing is recommended to link NHI with the quality of the provided service. Ensuring quality of care would ideally also require data on whether complications are managed in a certified Basic Emergency Obstetric and Neonatal Care (BEmONC) or CEmONC facility, and whether the facility is functioning properly. This information is currently lacking.

## Conclusions

The fact that women sought care and reached health facilities regardless of their insurance status provides an opportunity to prevent deaths by ensuring that all women receive timely and quality care. Timely care-seeking requires increased awareness of birth preparedness and complication readiness among women and their partners and families. This can be achieved by implementing health promotion strategies to encourage formal care-seeking, especially for complications during pregnancy and beyond the immediate postpartum period, which may be perceived as less severe than complications during delivery and within 24 hours postpartum. From a health financing perspective, the lower usage of insurance at the first formal provider, compared to the last provider seen, requires better understanding of barriers to using the insurance scheme. This information is critical to ensure timely care-seeking, and eventually, to leverage the effect of the nationally rolled-out health insurance in preventing maternal mortality and morbidity. To ensure quality care, the increasing demand due to the improved health financing should be met with balanced readiness of both primary care and hospitals, including the availability of competent human resources, essential drugs and properly-equipped facilities, supported by an effective referral system.

## Supporting information

S1 Table Causes of death by time of death, Jember District, 2017–2018. *Other causes: abortion, accidental exposure to smoke fire & flames, acute cardiac disease, acute respiratory infection including pneumonia, anemia of pregnancy, breast neoplasm, diarrheal diseases, digestive neoplasm, liver cirrhosis, other & unspecified non-communicable disease, other & unspecified maternal cause of death, road traffic accident ** Fisher’s exact test.
(DOCX)

S2 Table Wealth quartiles, place of death, and causes of deaths by insurance status, Jember District, 2017–2018. * Fisher’s exact test.
(DOCX)
