# Supplementary material for: Care-seeking and health insurance among pregnancy-related deaths: A population-based study in Jember District, East Java Province, Indonesia
Source: PLoS One. 2022 Mar 23;17(3):e0257278. doi: 10.1371/journal.pone.0257278 (PMC8942263; doi:10.1371/journal.pone.0257278)
Supplement: S1 Table — *Other causes: abortion, accidental exposure to smoke fire & flames, acute cardiac disease, acute respiratory infection including pneumonia, anemia of pregnancy, breast neoplasm, diarrheal diseases, digestive neoplasm, liver cirrhosis, other & unspecified non-communicable disease, other & unspecified maternal cause of death, road traffic accident ** Fisher’s exact test. (DOCX) [file pone.0257278.s001.docx]

**S1 Table. Causes of death by time of death, Jember District, 2017-2018**

| **Causes of death** | **Pregnancy**  **(n=25)** | **At delivery or within first 24 hr postpartum (n=37)** | **More than 24 hr postpartum (n=41)** | **p-value**** |
| --- | --- | --- | --- | --- |
|  | **n (%)** | **n (%)** | **n (%)** |  |
| Obstetric hemorrhage | 2 (8.0) | 29 (78.4) | 9 (22.0) | <0.001 |
| Pregnancy-induced hypertension | 8 (32.0) | 4 (10.8) | 9 (22.0) |  |
| Pregnancy-related sepsis | 7 (28.0) | 1 (2.7) | 9 (22.0) |  |
| Other and unspecified cardiac disease | 2 (8.0) | 3 (8.1) | 7 (17.1) |  |
| Other causes* | 6 (24.0) | 0 (0.0) | 7 (16.9) |  |

*Other causes: abortion, accidental exposure to smoke fire & flames, acute cardiac disease, acute respiratory infection including pneumonia, anemia of pregnancy, breast neoplasm, diarrheal diseases, digestive neoplasm, liver cirrhosis, other & unspecified non-communicable disease, other & unspecified maternal cause of death, road traffic accident

** Fisher’s exact test
