# Supplementary material for: Care-seeking and health insurance among pregnancy-related deaths: A population-based study in Jember District, East Java Province, Indonesia
Source: PLoS One. 2022 Mar 23;17(3):e0257278. doi: 10.1371/journal.pone.0257278 (PMC8942263; doi:10.1371/journal.pone.0257278)
Supplement: S2 Table — * Fisher’s exact test. (DOCX) [file pone.0257278.s002.docx]

**S2 Table. Wealth quartiles, place of death, and causes of deaths by insurance status, Jember District, 2017-2018**

|  | **Insured**  **(n=55)** | **Government aid**  **(n=24)** | **Uninsured**  **(n=24)** | **p-value*** |
| --- | --- | --- | --- | --- |
|  | **n (%)** | **n (%)** | **n (%)** |  |
| **Wealth quartile** |  |  |  | 0.127 |
| Quartile 1 (poorest) | 13 (23.6) | 3 (12.5) | 3 (12.5) |  |
| Quartile 2 | 15 (20.0) | 8 (33.3) | 6 (25.0) |  |
| Quartile 3 | 11 (27.3) | 1 (4.2) | 6 (25.0) |  |
| Quartile 4 (wealthiest) | 16 (29.1) | 12 (50.0) | 9 (37.5) |  |
| **Place of death** |  |  |  | 0.110 |
| Hospital | 43 (78.2) | 19 (79.2) | 15 (62.5) |  |
| Other health facility | 1 (1.8) | 3 (12.5) | 3 (12.5) |  |
| Home | 7 (12.7) | 0 (0.0) | 3 (12.5) |  |
| Enroute to hospital or facility | 4 (7.3) | 2 (8.3) | 3 (12.5) |  |

* Fisher’s exact test
